# Supplementary material for: Glycyrrhiza uralensis promote the metabolism of toxic components of Aconitum carmichaeli by CYP3A and alleviate the development of chronic heart failure
Source: PLoS One. 2022 Jun 27;17(6):e0270069. doi: 10.1371/journal.pone.0270069 (PMC9236245; doi:10.1371/journal.pone.0270069)
Supplement: S1 Table — (DOCX) [file pone.0270069.s001.docx]

Table 1 In vitro *t*_1/2_ data of AC, MA and HA in RLMs of pretreated rats (‾x ±*s*，*n*=5)

| Group | Dose  （g/kg） | *t_1/2_*（min） | | |
| --- | --- | --- | --- | --- |
|  |  | AC | MA | HA |
| Control | — | 256.90±9.52 | 173.32±4.33 | 263.25±5.70 |
| Phenobarbital | 0.08 | 170.57±6.49^**^ | 141.48±2.88^**^ | 198.11±5.66^**^ |
| Glycyrrhizae () | 0.33 | 189.03±3.00^*△^ | 156.33±2.02^*^ | 228.52±4.30^*^ |
| Glycyrrhizae | 1 | 179.33±5.26^**△△^ | 147.49±3.13^*^ | 203.94±6.00^**△▲^ |
| Glycyrrhizae | 3 | 239.16±8.25 | 155.37±7.10 | 239.16±8.25 |

^*^*P*＜0.05，^**^*P*＜0.01，vs Control；^△^*P*＜0.05，^△△^*P*＜0.01，vs Glycyrrhizae (3 g/kg);

^▲^*P*＜0.05，vs Glycyrrhizae (0.33 g/kg).
