# Supplementary material for: Glycyrrhiza uralensis promote the metabolism of toxic components of Aconitum carmichaeli by CYP3A and alleviate the development of chronic heart failure
Source: PLoS One. 2022 Jun 27;17(6):e0270069. doi: 10.1371/journal.pone.0270069 (PMC9236245; doi:10.1371/journal.pone.0270069)
Supplement: S2 Table — (DOCX) [file pone.0270069.s002.docx]

Tab. 2 In vitro intrinsic clearance ( CL_int_ ) of AC, MA and HA in RLMs of

pretreated rats (‾x ±*s*，*n*=5)

| Group | Dose  （g/kg） | CL_int_（μL/min/mg protein） | | |
| --- | --- | --- | --- | --- |
|  |  | AC | MA | HA |
| Control | — | 10.80±0.40 | 16.00±0.40 | 10.53±0.23 |
| Phenobarbital | 0.08 | 16.27±0.61^**^ | 19.60±0.40^**^ | 14.00±0.40^**^ |
| Glycyrrhizae () | 0.33 | 14.67±0.23^**△△^ | 17.73±0.23^*^ | 12.13±0.23^**^ |
| Glycyrrhizae | 1 | 15.47±0.46^**△△^ | 18.80±0.04^**^ | 13.60±0.40^**△▲^ |
| Glycyrrhizae | 3 | 11.60±0.40 | 17.87±0.83 | 11.60±0.40 |

^*^*P*＜0.05，^**^*P*＜0.01，vsControl；^△^*P*＜0.05，^△△^*P*＜0.01，vsGlycyrrhizae (3 g/kg);^▲^*P*＜0.05，vsGlycyrrhizae (0.33 g/kg).
