# Supplementary material for: Glycyrrhiza uralensis promote the metabolism of toxic components of Aconitum carmichaeli by CYP3A and alleviate the development of chronic heart failure
Source: PLoS One. 2022 Jun 27;17(6):e0270069. doi: 10.1371/journal.pone.0270069 (PMC9236245; doi:10.1371/journal.pone.0270069)
Supplement: S3 Table — (DOCX) [file pone.0270069.s003.docx]

Table 3 Metabolites A1, A2, M1 and H1 in vivo (‾x ±*s*，*n*=3)

| Group | Dose  (g/kg) | Metabolites | | | |
| --- | --- | --- | --- | --- | --- |
|  |  | A1 | A2 | M1 | H1 |
| Control | — | 4.153±0.6501 | 8.147±0.7337 | 3.86±0.9948 | 3.400±0.6525 |
| Phenobarbital | 0.08 | 7.367±0.9567^**^ | 12.900±1.4726^**^ | 6.833±1.2454^**^ | 4.427±0.7146^**^ |
| Glycyrrhizae () | 0.33 | 5.273±1.0552^**▲^ | 10.520±1.1930^**^ | 4.407±0.8697 | 3.627±0.8163 |
| Glycyrrhizae | 1 | 5.567±0.8797^**△^ | 10.480±1.7624^**△^ | 5.313±1.8334^*^ | 4.013±0.6578^*△^ |
| Glycyrrhizae | 3 | 4.653±0.8348 | 9.460±1.5127^*^ | 3.867±0.9029 | 3.327±0.6296 |

^*^*P*＜0.05，^**^*P*＜0.01，vs Control；^△^*P*＜0.05，^△△^*P*＜0.01，vs Glycyrrhizae (3 g/kg);

^▲^*P*＜0.05，vs Glycyrrhizae (0.33 g/kg).
