# Supplementary material for: Glycyrrhiza uralensis promote the metabolism of toxic components of Aconitum carmichaeli by CYP3A and alleviate the development of chronic heart failure
Source: PLoS One. 2022 Jun 27;17(6):e0270069. doi: 10.1371/journal.pone.0270069 (PMC9236245; doi:10.1371/journal.pone.0270069)
Supplement: Attachment — Submitted filename: Response to Reviewers.docx [file pone.0270069.s006.docx]

May 6, 2022

Dear Reviewer:

First of all, we thank you for your thoughtful suggestions and insights. The manuscript has benefited from these insightful suggestions. The manuscript ID is PONE-D-22-05670.

The manuscript has been rechecked and the necessary changes have been made in accordance with the reviewers’ suggestions. The responses to all comments have been prepared and included in the main text.

**Reviewer 1**

EVALUATION: In this manuscript, the metabolism of AC, MA and HA in Aconitum was intervened by regulating CYP450 with Glycyrrhiza, and the protective effect and mechanism of Glycyrrhiza combined with Aconitum on CHF were explored from the perspective of neuroendocrine system and myocardial energy metabolism in rats with heart failure, providing an in-depth basis for the pharmacological study of the compatibility of Aconitum carmichaeli with licorice. So I think that the manuscript is innovative and can be published in this journal.

Before this manuscript is accepted, there are some errors that need to be amended.

1. In the statistical analysis, the number of samples ( n ) is not well pointed out, which makes it difficult to ensure the statistical significance of the data.
2. In Figs 1B and 1D, there are still two unmarked peaks. why they are not explored?
3. In the discussion section, the sentence “we found that the metabolic rates of AC, MA and HA induced by different doses of Glycyrrhiza were different (medium dose group > low dose group > high dose group)” was pointed out. Therefore, intergroup comparisons should be added in statistical analysis.
4. The format of the manuscript is chaotic and needs to be corrected. Image clarity is not enough, it is difficult to see the data, such as Fig 3, Fig 6 and so on .

**Response:** Thank you for your advice.

1. In the statistical analysis, n=3 in vitro, n=5 in vivo.
2. The two unmarked peaks in figures 1B and 1D contained both blank control samples and standard samples. They are considered to be impurities and did not affect the final result.
3. In the study on the metabolic rate of AC, MA and HA induced by licorice, we further detected the changes of metabolic stability parameters (*t*_1/2_ and CL_int_) of diester alkaloids in rat liver microsomes in each group (Supplementary Table 1 and 2) and carried out statistical analysis between groups. At the same time, the differences in the production of A1, A2, M1 and H1 metabolites in different groups were statistically analyzed (Supplementary Table 3).
4. Thank you for your advice. We have corrected the format of the manuscript. Because the columns in Figure 3 and the lines in Figure 6 were dense, we chose to upload them in the form of a single graph, so that you could clearly see all the datas in the graph. In Figure 5, the trend line color of each group was changed to make it clearer. And other pictures could be enlarged to see clearly.

All the above changes will be presented at the revised.

Thank you for your consideration. I look forward to hearing from you.

Sincerely,

Dr. Su Tu

Department of emergency, the Affiliated Wuxi NO.2 People's Hospital of Nanjing Medical University, No. 68, Zhongshan Road, Wuxi, Jiangsu 214000, PR China

Tel: +86-0510-68562222

Email: tusuwxey@126.com
